# Rats Exposed to Excess Sucrose During a Critical Period Develop Inflammation and Express a Secretory Phenotype of Vascular Smooth Muscle Cells

**DOI:** 10.3390/metabo14100555

**Published:** 2024-10-17

**Authors:** Verónica Guarner-Lans, Elizabeth Soria-Castro, Agustina Cano-Martínez, María Esther Rubio-Ruiz, Gabriela Zarco, Elizabeth Carreón-Torres, Oscar Grimaldo, Vicente Castrejón-Téllez, Israel Pérez-Torres

**Affiliations:** 1 Department of Physiology, Instituto Nacional de Cardiología “Ignacio Chávez”, Juan Badiano 1, Sección XVI, Tlalpan, Mexico City 14080, Mexico; veronica.guarner@cardiologia.org.mx (V.G.-L.); agustina.cano@cardiologia.org.mx (A.C.-M.); esther.rubio@cardiologia.org.mx (M.E.R.-R.);; 2 Department of Cardiovascular Biomedicine, Instituto Nacional de Cardiología “Ignacio Chávez”, Juan Badiano 1, Sección XVI, Tlalpan, Mexico City 14080, Mexico; elizabethsoria824@gmail.com; 3 Department of Pharmacology, Instituto Nacional de Cardiología “Ignacio Chávez”, Juan Badiano 1, Sección XVI, Tlalpan, Mexico City 14080, Mexico; 4 Department of Molecular Biology, Instituto Nacional de Cardiología “Ignacio Chávez”, Juan Badiano 1, Sección XVI, Tlalpan, Mexico City 14080, Mexico; juana.carreon@cardiologia.org.mx

**Keywords:** hypertension, sucrose ingestion, critical window, synthetic phenotype, vascular smooth muscle cells, inflammation, cyclooxygenase 2, toll-like receptor 4

## Abstract

Background: Neonatal rats that receive sucrose during a critical postnatal period (CP, days 12 to 28) develop hypertension by the time they reach adulthood. Inflammation might contribute to changes during this period and could be associated with variations in the vascular smooth muscle (VSMC) phenotype. Objective: We studied changes in inflammatory pathways that could underlie the expression of the secretory phenotype in the VSMC in the thoracic aorta of rats that received sucrose during CP. Methods: We analyzed histological changes in the aorta and the expression of the COX-2, TLR4, iNOS, eNOS, MMP-2 and -9, and β- and α-actin, the quantities of TNF-α, IL-6, and IL-1β using ELISA, and the levels of fatty acids using gas chromatography. Results: The aortic wall presented disorganization, decellularization, and wavy elastic fibers and an increase in the lumen area. The α- and β-actin expressions were decreased, while COX-2, TLR4, TNF-α, and the activity of IL-6 were increased. Oleic acid was increased in CP in comparison to the control group. Conclusions: There is transient hypertension at the end of the CP that is accompanied by inflammation and a change in the phenotype of VSMC to the secretory phenotype. The inflammatory changes could act as epigenetic signals to determine the development of hypertension when animals reach adulthood.

## 1. Introduction

The aorta, a conductance artery, is the largest artery in animals and humans, and modifications in its structure and/or function alter the cardiovascular system [1]. Both changes in conductance and resistance arteries contribute to hypertension [2]. The aortic wall has three layers: the adventitia; the media, comprising the vascular smooth muscle cells (VSMCs), collagen, and elastic fibers (EF); and the intima (endothelium) [3]. The mechanical properties of the aorta depend on the amount of the main components of these layers, and on the spatial organization and mechanical interactions between them [4]. Changes in the phenotype of VSMCs in the media are often present in conductance and resistance arteries, and they underlie increases in the stiffness of the vessel, leading to hypertension. Two types of phenotypes are described in VSMCs: contractile and secretory (or synthetic). Markers of differentiation into the secretory phenotype include (a) increased cell size; (b) increased extracellular matrix production, which involves metalloproteinases (MMPs) and increased collagen III and fibronectin; (c) increased migration; and (d) decreased contractile protein expression, including smooth muscle actin and increased osteopontin [5]. However, the control of VSMC differentiation/maturation, and the regulation of its responses to changing environmental cues, is complex and depends on the cooperative interaction of many factors and signaling pathways [6]. Changes in the phenotype of VSMCs have been extensively described in conductance and resistance arteries [7]. Sucrose ingestion during critical periods (CP) of vessel development might induce inflammation and determine a change in the VSMC phenotype. Changes in the expression of MMPs during remodeling of the VSMC phenotype may also contribute to overexpression of inflammatory cytokines [8].

Previous papers have shown that changes in diet, mainly low protein [9] or high salt ingestion [9], during gestation and lactation, underlie the development of hypertension during adulthood without exploring the role of inflammation. These periods range from 1 to 3 months. However, the effect of a shorter time lapse of only 16 days of a modified diet during the last days of lactation and the first days after weaning (rat postnatal days 12 to 28) also results in hypertension when the rats reach adulthood [10]. This stage, therefore, constitutes a CP of vessel differentiation [11]. During this CP, the rodent diet changes from being rich in fat to being rich in carbohydrates, and the pancreas undergoes important maturation [12]. The maturation of the pancreas is accompanied by changes in glucose and insulin concentrations in the plasma, which modifies vascular reactivity [13] and may induce inflammation. Variations in blood glucose and insulin concentrations are related to oxidative stress (OS), inflammation, and changes in redox signaling [14]; these might lead to changes in the VSMC phenotype in this CP of development [15,16]. Therefore, changes induced by a modified diet might induce inflammation during the critical window, which might program VSMCs to the secretory phenotype.

Inflammatory mediators can override homeostatic processes, including the expression of MMPs that lead to changes in the phenotype of VSMCs, thus determining the mechanical function of the vessels [17]. Tumor necrosis factor alpha (TNF-α) causes a fundamental change from a contractile to a secretory phenotype of VSMCs. This switch enhances the proliferation and production of extracellular matrix proteins, which are associated with hypertrophy of the media [18]. TNF-α and IL-1β induce the growth and/or migration of VSMCs and stimulate the production of chemokines, leading to a ‘proinflammatory’ phenotype of VSMCs, which then secrete and express other proinflammatory cytokines, such as IL-6 and cell adhesion molecules [19]. Additionally, elevated exposure to glucose, such as that consumed in drinking water during a critical period, might induce hypertriglyceridemia [20]; this condition promotes the secretion of many cytokines in human white blood cells and possibly in other tissues and cells [21], which in turn modulates MMP activity.

On the other hand, cyclooxygenase-2 (COX-2) is an enzyme largely responsible for causing inflammation, and its inhibition is associated with hypertension. It is an inducible enzyme expressed by inflammatory cells and blood vessels [22]. This enzyme metabolizes arachidonic acid (AA) to prostaglandin E_2_ (PGE_2_), which is proinflammatory and has vasoconstrictor effects [23]. Another enzyme that contributes to inflammation is the inducible nitric oxide synthase (iNOS), which catalyzes the conversion of L-arginine to L-citrulline and nitric oxide (NO); the excess NO acts as a proinflammatory agent [24]. Both COX-2 and iNOS have synergistic actions promoting and maintaining key physiological functions such as vasomotion. There is also cross-talk between NO and vasoconstrictor prostanoids, which decreases eNOS expression. However, the details of the cross-talk between prostanoids and the iNOS–NO system, and on the eNOS pathway, remain unknown [25].

Inflammation can also be induced by fatty acids, such as oleic and arachidonic acid (AA), by activating toll-like receptors (TLR); in particular, the TLR4 signaling pathway. TLR4 also increases COX-2 expression [26]. Oleic acid elevation in plasma might also contribute to a decrease in eNOS, and this can, in turn, increase systolic blood pressure (SBP) [27].

Therefore, the aim of this paper was to study changes in the VSMC phenotype and in the expression of MMPs in rats that received sucrose at the end of the CP, before compensatory mechanisms are established. We tested the possible involvement of the COX-2 and TLR4 pathways, which are related to iNOS and eNOS. We also studied the levels of inflammatory mediators in this group of rats.

## 2. Materials and Methods

### 2.1. Animals and Experimental Groups

Experiments in animals were approved by the Laboratory Animal Care Committee of our institution and were conducted in compliance with our institution’s ethical guidelines for animal research (INCAR protocol number # 20-1147).

**Figure 1:**
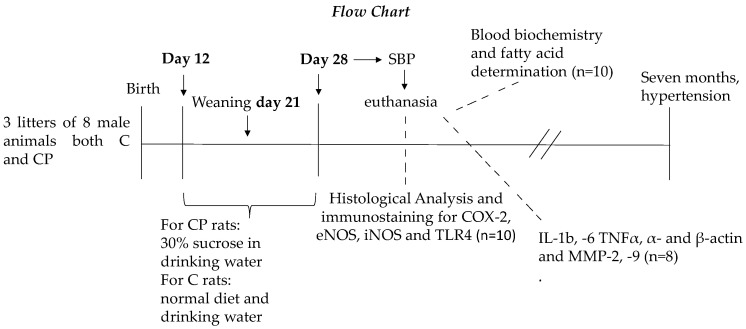
Flow chart for the management of the experimental animals.

A group of rats was given 30% sucrose in their drinking water during the CP of vessel development (postnatal days 12 to 28); the control (C) group received a normal diet and drinking water. The animals in both groups were of the same age. Pups from 8 gestating mother rats were used. The litters were adjusted to 8 male pups per mother from the moment of birth, and each mother was housed in a separate cage. The mothers were fed Purina 5001 rat chow (Richmond, IN, USA) ad libitum and were kept under controlled temperatures and a 12:12-h light-dark cycle. Pups were fed milk from their mothers on the first days of the CP, and then were fed a mixture of milk and rat chow until weaning. Pups were separated from the mothers on postnatal day 21, and from that day on, they were only fed rat chow. The 8 neonatal rats from each litter were kept in independent cages. Rats from at least 3 litters of 6 male animals for the C and CP groups were used for pressure measurement, and other rats from at least 3 litters of 8 male animals for the CP and C groups were used for serum and tissue determinations. All pups were sacrificed on postnatal day 28. Figure 1 shows the flow chart for the proposed study.

### 2.2. Blood Pressure Measurement and Sacrifice

For SBP determination, six 28-day-old rats from 3 litters of control and CP that were previously fasted for 12 h were weighed and intraperitoneally anesthetized with 50 mg/kg of sodium pentobarbital and allowed (Anestesal; Pfizer, Mexico City, Mexico) to reach a state of surgical anesthesia. An intratracheal tube was placed for respiration. A catheter filled with Hartmann solution—heparin (3:1)—was placed in the left cranial carotid artery and connected to a blood pressure transducer to a previously calibrated polygraph VR-6 simultrance recorder (Model M4-A, Electronics for Medicine/Honeywell, White Plains, NY, USA). Five minutes of recuperation after surgery were allowed before the register was obtained. The mean of five independent determinations was calculated.

Another group of rats was sacrificed by decapitation after overnight fasting (12 h). The blood was collected, and the serum was separated using centrifugation at 600× *g* for 15 min at room temperature and stored at −70 °C until needed. Thoracic aortas were obtained and cleaned of blood and adipose tissue. Pools of 3 aortas from C and CP rats were made for western blot analysis. Aortas were chosen because changes in phenotype have been described in conductance arteries [7]. Moreover, the amount of tissue from resistance arteries is difficult to obtain in neonatal rats of this age.

### 2.3. Biochemical and Physiological Determinations

Glucose concentration was assayed using an enzymatic SERA-PAKR Plus from Bayer Corporation (Bayer Corporation, Sées, France). Serum insulin was determined using a commercial radioimmunoassay (RIA) specific for rats (Linco Research, Inc., St. Charles, MO, USA); its sensitivity was 0.1 ng/mL, and the intra- and inter-assay coefficients of variation were 5 and 10%, respectively. The HOMA-IR was calculated from the fasting glucose and insulin concentrations. The homeostasis model assessment of insulin resistance (HOMA-IR) is used as a physiological index of insulin resistance, and it is determined from the fasting glucose and insulin concentrations using the following formula: (insulin (µU/mL) × glucose (in mmol/L)/22.5). Triglycerides (TGs) were determined using commercially available procedures (Ran-dox, Laboratories LTD, Antrim, UK).

### 2.4. Histological Analysis

Cross sections (5 µm) of C and CP aortas were processed using paraffin inclusion. They were stained using the conventional method for Hematoxylin-Eosin (HE) staining or immunolocalization for COX-2, TLR4, iNOS, and eNOS. For HE staining, 10× 20×, or 25× images were obtained around the ring from which the total image of the ring was reconstructed (*n* = 10), superimposing the photographs at the coincidence points. Images were obtained using an Olympus BX51 microscope, with an integrated camera [Q-IMAGING, Micropublisher 5.0 RTV (Real-Time Viewing)] coupled to Image-Pro Premier software, version 9.0, (Media Cybernetics). The average value of the wall thickness was obtained from 20 measurements at equidistant points from each ring (*n* = 10). Total lumen area (*n* = 10) and total wall area (*n* = 10) were measured. From the same sections with HE staining, images in a gray tone were acquired using a Floid Cell Imaging Station (Life Technologies, Santa Clara, CA 95051, USA), with the color channel in relief phase, since changes can be more clearly appreciated. Approaches are presented in which the differences in the structure of the aortic wall of group C and CP are distinguished in greater detail.

For the immunostaining of COX-2, eNOS, iNOS, and TLR4, the aortic rings of each rat were preserved in 10% formalin in a 1:20 ratio. The immunohistochemistry was processed according to the conventional histological technique. The samples were incubated with the primary monoclonal antibodies at a final dilution of 1:20 for all antibodies: COX-2 sc-19999, IgG_1_; iNOS (c-11) sc-7271, IgG_1k_; eNOS (a-9) sc-376751, IgG_2ak_; and TLR4 (25) sc-293072. The staining was revealed with DAB (3’3’-diaminobenzidine) and contrasted with hematoxylin. The histological sections were analyzed using a Carl Zeiss light microscope (66300 Model) equipped with a 9-megapixel Cool SNAP-Pro digital camera at 25× magnification. The photomicrographs were analyzed using densitometry in Sigma Scan Pro 5 Image Analysis software (Systat Software Inc., San Jose, CA, USA), and the parameters of analysis in the software were adjusted and remained constant for each of the antibodies. An average of five sections of endothelium and the muscular media layer in each sample was examined. The density values are expressed as pixel area units.

### 2.5. Western Blott Analysis (MMP-2, MMP-9, SMA, β-Actin)

Aortas were homogenized in lysis buffer plus protease/phosphatase inhibitor cocktail. The homogenate was centrifuged at 14,000 rpm for 15 min at 4 °C; the supernatant was separated and stored at −70 °C until use. The Bradford method was used to determine the total protein [26].

Protein (50 μg) was separated on an SDS-PAGE gel and transferred to polyvinylidene difluoride (PVDF) membranes. Blots were blocked for 1 h at room temperature using Tris-buffered saline (TBS)-0.01% Tween (TBS-T 0.01%) plus 5% nonfat milk. The membranes were incubated overnight at 4 °C with rabbit primary polyclonal antibodies: β-Actin (sc-81178), metalloproteinase 2 (MMP-2; sc-13595), metalloproteinase 9 (MMP-9; sc-393859), and Smooth Muscle Actin (SMA; sc-53142), all from Santa Cruz Biotechnology (Santa Cruz, CA, USA). All blots were incubated with glyceraldehyde 3-phosphate dehydrogenase (GAPDH; sc-365062) antibodies as a loading control. Images from films were digitally obtained using a GS-800 densitometer with the Quantity One software (Bio-Rad Laboratories, Inc., Hercules, CA, USA) and reported as arbitrary units (AU).

### 2.6. Total Fatty Acid (TFA) Determination

For the extraction and derivatization of fatty acids (FA), 100 µL of serum from C and CP animals was used according to the method described by Folch [28], in the presence of 50 µg of margaric acid (C17:0) as an internal standard. Then, 1 mL of saline solution (0.09%) was added and mixed for 15 s, and then 2 mL of a methanol chloroform mixture (2:1 vol/vol) plus 0.002% BHT were added and centrifuged at 3000 rpm for 5 min. This step was repeated twice, and the organic phase was recovered and evaporated under a gentle current of nitrogen. FAs were trans-esterified to their FA methyl esters by heating them at 90 °C for 2 h with 2 mL of methanol plus 0.002% BHT, 40 µL of H_2_SO_4_, and 100 µL of toluene. Afterward, 1 mL of saline solution and 4 mL of hexane were added, and the mixture was centrifuged at 3000 rpm for 5 min. The hexane phase was recovered and evaporated under a gentle current of nitrogen. The evaporated residue containing the FA was suspended in 100 µL of hexane, and 4 µL was injected into the chromatograph. The FA methyl esters were separated and identified using gas chromatography-FID in a Carlo Erba Fratovap 2300 chromatograph (CARLO ERBA Reagents GmbH, Denzlinger Str. 27 79312 Emmendingen) equipped with a capillary column packed with the stationary phase HP-FFAP (description: 30 m length × 0.320 mm diameter × 0.25 µm film) and fitted with a flame ionization detector at 210 °C, with helium as the carrier gas at a flow rate of 1.2 mL/min. The areas under the peaks were calculated using Chromatograph software version 1.1 coupled to the gas chromatograph. The identification of each FA methyl ester was made by comparing its retention time with its corresponding standard.

### 2.7. Determination of Inflammatory Mediators

The ELISA kit ab100768 -IL-1β for Rat, the ELISA Kit ab 100772 Rat IL-6, and ELISA Kit ab100785 TNF alpha Rat Simple Step were used in a double-antibody sandwich ELISA method to measure inflammatory cytokines in rat serum. The anti-rat IL-1β, IL-6, and TNF-α antibodies were coated on an enzyme plate. During the experiment, the rat IL-1β, IL-6, and TNF-α, in the sample or standard product, were bound to the coated antibody, and the free components were washed away. Biotinylated anti-rat IL-1β, IL-6, and TNF-α antibodies, as well as horseradish peroxidase labeled with streptavidin, were successively added. The anti-rat IL-1β, IL-6, or TNF-α antibodies were bound to the coated antibody for rat IL-1β, IL-6, and TNF-α, and the biotin specifically bound to avidin to form an immune complex, while the free components were washed away. Color substrate (TMB) was added, and it became blue under the catalysis of horseradish peroxidase then turned yellow with the addition of the termination solution. The concentration of IL-1β, IL-6, and TNF-α in the sample was proportional to the OD450 value. The concentration of IL-1β, IL-6, and TNF-α in the sample was calculated by drawing standard curves.

### 2.8. Statistical Analysis

Results are expressed as mean ± standard errors. The type of distribution was evaluated using the Shapiro–Wilkes test. Statistical analysis was performed using Student’s *t* test with the Sigma Stat program version 15 (Jandel Scientific, 405 Waverley St, Palo Alto, CA 94301, USA). Differences were considered statistically significant when *p* < 0.05.

## 3. Results

### 3.1. Body Variables and Blood Pressure

Body variables were determined since they might be modified by the exposure to sucrose in drinking water during the CP of vessel development, even if it only lasted 12 days. Body weight was significantly diminished in 28-day-old rats that received sucrose from postnatal day 12. We have previously reported that water and food intake was diminished by the addition of sucrose in drinking water, but total Kcal obtained from the diet plus the sucrose in the drinking water was increased [29,30]. Visceral adipose tissue was not modified. There were no significant changes in serum glucose and insulin concentrations. Therefore, the HOMA-IR index was not modified (Table 1).

The data obtained from the determination of FA show that the percentage of oleic acid was increased in the CP group with respect to the C group, while linoleic acid was decreased, with significant changes (*p* < 0.05) (Table 1).

Mean SBP was significantly increased in 28-day-old rats that received sucrose in comparison to C rats (*p* < 0.05, Table 1).

### 3.2. Changes in the Histology of the Aortic Rings from C and CP Rats

**Figure 2:**
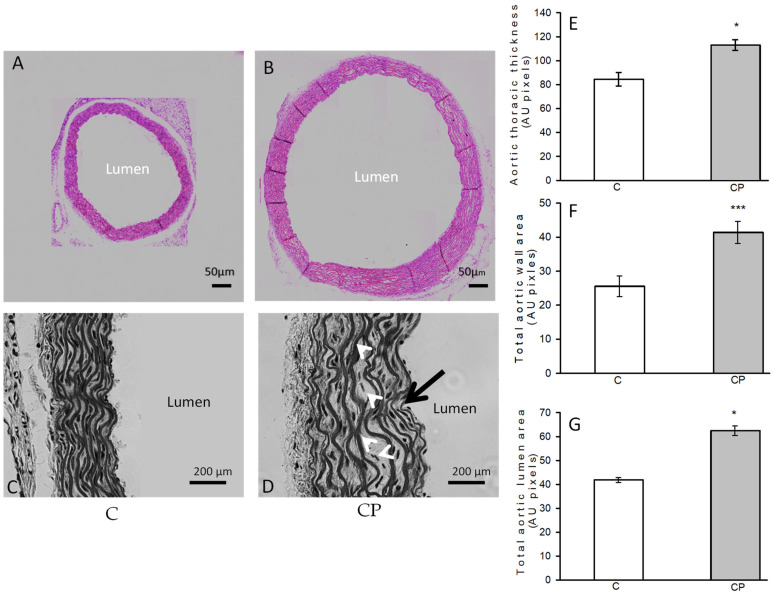
Histological changes of the aortic rings from control rats receiving the normal diet (**A**,**C**) and rats exposed to sucrose in the drinking water during the CP (**B**,**D**). The pink-stained images were acquired from histological sections stained with HE using a bright field microscope coupled to a camera (see Section 2). From the same sections with HE staining, images in a gray tone were acquired with the relief phase color channel for close-ups in which the differences in the structure of the aortic wall between the two groups are distinguished in more detail. In the lower-right gray image, the locations of undulations (black arrow) and aneurysms (white arrows) are indicated. On the right side of the image, the graphs of the comparison of the measurements of the thickness (**E**) and the total area of the wall (**F**), as well as the total area of the aortic lumen (**G**), are shown. Values represent the mean ± standard error (*n* = 6), * *p* = 0.01, *** *p* = 0.001 (Figure 1).

The closeups showed differences in the structure of the aortic wall between the C group and CP, distinguished in more detail (Figure 2A–D). A defined and organized structure of the aortic wall was identified in the controls. In contrast, the structure of the aortic wall of the CP aortas presented disorganization, wavy EF (1.8%) without reaching a statistically significant difference, and alterations in the smooth muscle, in which decellularization and aneurysms were observed, mainly in the central part of the media. The values of total lumen area, total area and aortic wall thickness are greater in the CP group when compared to control group (Figure 2E–G, respectively).

### 3.3. Expression of COX-2, eNOS, iNOS and TLR4

**Figure 3:**
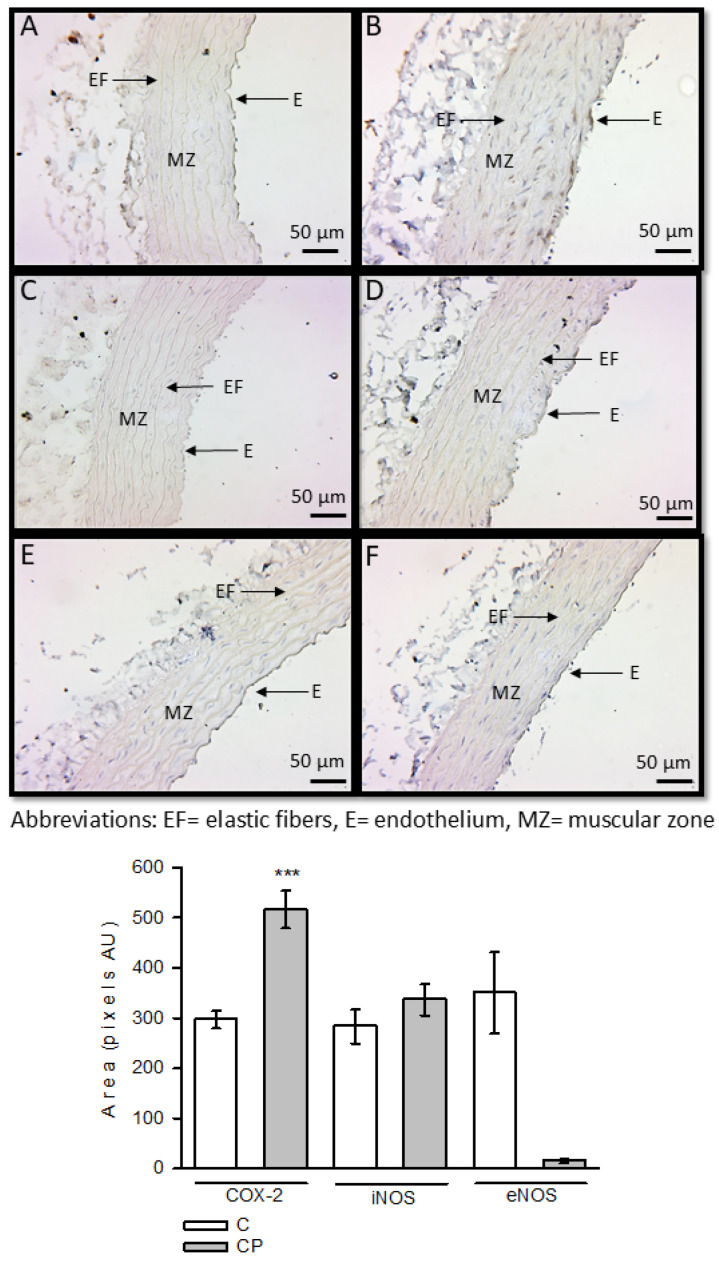
Immunohistochemistry for COX-2, iNOS, and eNOS in control aortas and aortas from rats that received sucrose during the CP. Panels (**A**,**C**,**E**) (COX-2, iNOS, and eNOS in the C group, respectively) and panels (**B**,**D**,**F**) (COX-2, iNOS and eNOS in the CP group, respectively). *** *p* < 0.001. Values represent the mean ± SE, *n* = 10, per group. Abbreviations: EF = elastic fibers, E = endothelium, MZ = muscular zone.

Expression of these enzymes and the TLR4 receptor was determined using immunohistochemistry. COX-2 was overexpressed in aortas from CP rats, with a significant difference (*p* < 0.001). However, iNOS and eNOS showed no statistical changes in any group (Figure 3).

**Figure 4:**
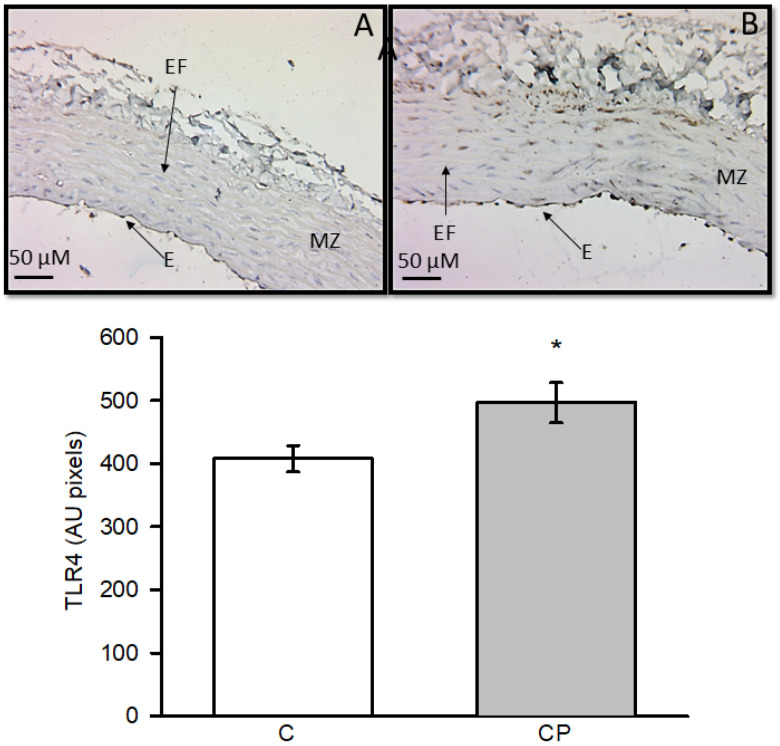
Immunohistochemistry for TLR4 in control aortas (**A**) and aortas from rats that received sucrose during the CP (**B**). * *p* < 0.03 Values represent the mean ± SE (*n* = 10 per group). Abbreviations: EF = elastic fibers, E = endothelium, MZ = muscular zone.

Expression of TLR4 was elevated in aortas from CP rats with a statistically significant difference (*p* < 0.03, Figure 4).

### 3.4. Inflammatory Cytokines IL-1β, IL-6, and TNF-α

**Figure 5:**
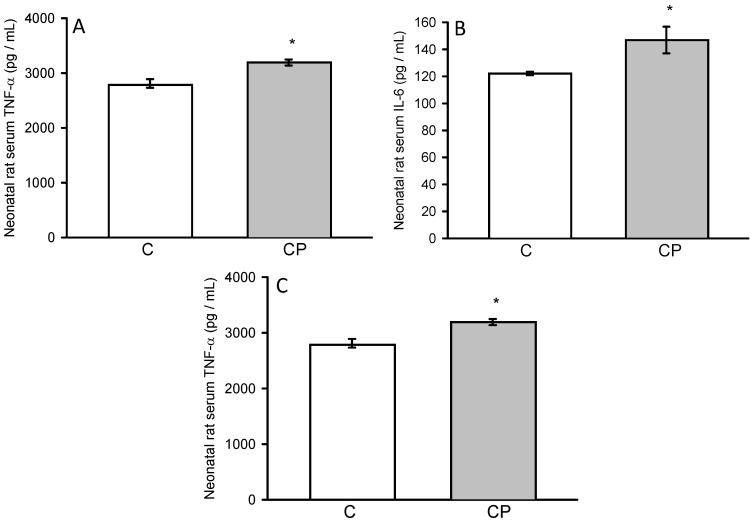
Concentrations of interleukins IL-1β (**A**), IL-6 (**B**), and TNF-α (**C**) in serum from control and CP rats.. Values represent the mean ± SE (n = 8 per group), * *p* < 0.05.

Activity of TNF-α and IL-6 showed an increase in serum from CP rats (*p* < 0.05, Figure 5), while IL-1β showed a tendency to be elevated in comparison with control rats.

### 3.5. VSMC Phenotype and Expression of Metalloproteinases and Immune Mediators

**Figure 6:**
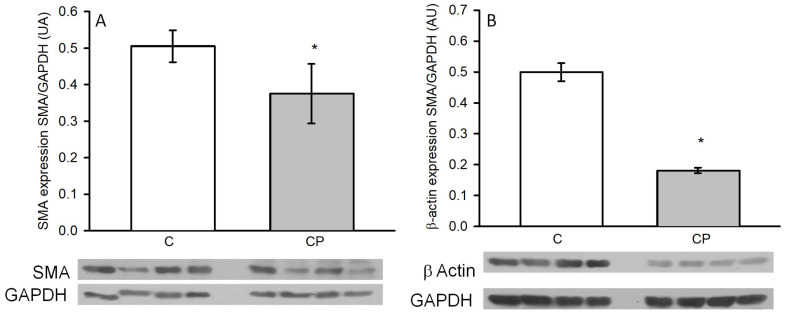
Changes in the expression of SMA (**A**) and β-actin (**B**) in thoracic aortas from C rats and CP rats. Values represent the mean ± SE (*n* = 8 animals per group). * *p* < 0.05. Representative western blot images are included in the lower panel. AU refers to arbitrary units, which are determined as the relative density of the band of the protein of interest in relation to the control of charge protein (GAPDH).

α-actin (smooth muscle α-actin, SMA), a marker of the contractile phenotype of VSMCs, was determined in the aortas by the western blot technique, and it was significantly decreased in rats exposed to sucrose in drinking water during the CP, which might indicate that the aortas show a secretory phenotype. The expression of the cytoplasmic or non-contractile β-actin was also diminished in rats from the CP group in comparison to the C group (*p* < 0.05, Figure 6).

**Figure 7:**
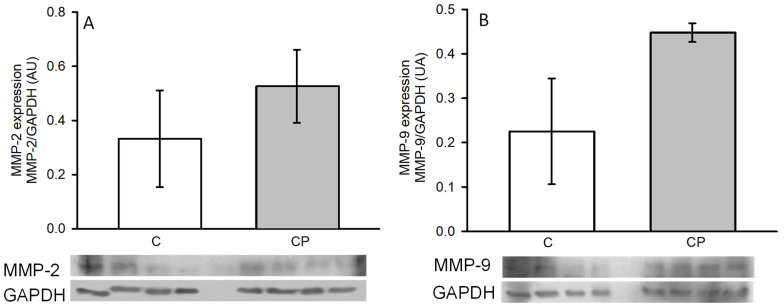
Changes in the expression of MMP-2 (**A**) and -9 (**B**) in thoracic aortas from control rats receiving the normal diet (C) and rats receiving sucrose during the critical period (CP). Values represent the mean ± SE, *n* = 8 animals per group. Representative western blot images are included in the lower panel. AU refers to arbitrary units which are determined as the relative density of the band of the protein of interest in relation to the control of charge protein (GAPDH).

The expressions of MMP-2 (A) and -9 (B), which are involved in the remodeling of arteries during the development of hypertension, through the breakage of the fibrous material surrounding the cells, tended to be elevated in aortas from CP rats in comparison to those of the control rats without reaching a statistically significant difference (Figure 7).

## 4. Discussion

Changes in the diet during early development program organisms to develop hypertension when they grow into adulthood [11]. Therefore, epigenetic cues might appear at this stage that result in the development of this disease later in life. In this sense, essential hypertension, like other heart diseases, has been reported to be programmed since childhood [29,30,31,32,33]. Moreover, there is a possible association between SBP levels in childhood and hypertension in the adult [34]. Therefore, changes occurring at early stages of life might predispose to the development of diseases in adult life, rendering important the study of critical periods [35]. In this study, we evaluated the inflammatory pathways that may lead to morphological changes in the aortas of rats receiving sucrose during a CP of vascular development.

We previously reported that the effect of exposure to sucrose in the drinking water during a CP of vessel development that lasts for only 16 days (from postnatal day 12 to 28), comprising a period in which pups are still suckling milk from the mother and a period in which they are feeding independently, results in hypertension in adulthood. The increase in SBP was accompanied by elevated NO levels, reduced expression of eNOS, and OS in the thoracic aortas [10].

When studying the changes caused by the exposure to sucrose in drinking water at the end of the critical period that might underlie the epigenetic cues, we corroborated the previous findings from our group in which we found that pups that received sucrose during this CP had a decreased body weight, and there were no changes in serum glucose or insulin while TG levels were increased. The decrease in body weight in rats that received sucrose, even when the diet contained more Kcal, could be due to excess activity of the pups, as previously discussed [29]. The lack of changes in glucose and insulin concentration might be the consequence of the short time of exposure to sucrose. The increase in TG results from the high exposure to sucrose [28].

At this stage, SBP was elevated, and there was a diminished eNOS expression in the aorta. This elevation of SBP, was also found in the present study, is transient, since in a previous paper, we reported that at 4 months of age, rats receiving sucrose had a comparable SBP as C rats, and that at this point of development, arterial pressure began to rise, leading to hypertension at 6 months of age [10]. Therefore, this increase at the end of the CP might set an epigenetic cue that is lost during the next months of development but programs the arterial contraction in adulthood [34].

Remodeling of vessels, which might underlie hypertension in adults, is characterized by an elevation in the stiffness of large arteries, which decreases their ability to modify the pulsatile pressure to a continuous pressure and flow in arterioles. Intrinsic stiffness and arterial geometry of the vessel underlie arterial compliance [35,36]. Cellular processes that determine these characteristics include altered VSMC growth, migration, differentiation, and increased extracellular matrix abundance [37]. Regarding changes in the phenotype of the VSMCs when rats receive sucrose during the CP window, we previously reported morphological changes, a decreased expression of α-actin, and, surprisingly, a decrease in MMP-2 and -9 expression, suggesting that compensatory mechanisms to the changes in the aortas at the early stage were activated [28]. The surprising finding of a MMP decrease, which contradicts the previous expected results of an increase in MMP expression, motivated the study on the possible increase of the expression of these proteins earlier, possibly at the end of the altered diet period, during which the arteries are programmed to develop the altered phenotype underlying the development of hypertension in the adult. It has been previously reported that the expression of active MMPs is absent or very low in mature and quiescent vessels, in contrast to the high expression, secretion, and activation of these enzymes found in the tissues undergoing vascular remodeling, which might be happening at the end of the critical period of vessel development.

In the present study, we found that the morphology of the aortas from rats that received sucrose during the CP is altered and that they show eccentric hypertrophy, which is characterized by dilatation of the lumen of the artery (Figure 1). Eccentric hypertrophy is due to an increase in the overall size of myocytes, which results from an in-series increase in contractile proteins. Eccentric hypertrophy is usually the result of volume overload and leads to diastolic stress [38]. In our model, this type of hypertrophy was accompanied by an increase in smooth muscle mass. Vascular stiffness results from fibrosis, which was not observed in the aortas from our experimental groups, and extracellular matrix remodeling. Fibrosis was probably not found due to the short lapse of time that comprises the critical period of vascular function (only 12 days).

It is possible that some of the structural changes observed in the images shown in Figure 1 are associated with the presence of hemodynamic shear stress that could be present in the animals that received sucrose during the CP. Shear stress, the frictional force acting on the inner surface of blood vessels in the direction of blood flow, is linked to elevated blood pressure. Shear stress may be involved in the development of changes in the endothelium and may regulate vascular caliber, cell proliferation, and inflammation of the vessel wall (vascular remodeling) in hypertensive patients and in several animal models, leading to alterations in the structure and function of blood vessels [39]. Furthermore, the presence of aneurysms observed in the aortas of CP animals (marked with white arrows in the images in Figure 1) also coincides with what has been reported by other works that indicate that shear stress favors the development of aneurysms [40,41]. Although we did not evaluate changes in resistance vessels, since their size in neonatal rats is very small, changes in VSMCs in these vessels might resemble those in conductance vessels. However, to undertake another study in these vessels could be of interest.

Histological changes in the aortas and alterations in the VSMC phenotype of the vessels at the end of the exposure to sucrose may be due to underlying inflammatory processes that have not been evaluated. The role played by inflammation in the change of the VSMC phenotype and the possible inflammatory pathways involved have not been studied, and they are the aim of the present paper. High levels of glucose and insulin induce an inflammatory process [15]. Although the serum levels of these variables were not increased at the end of the CP, there is exposure to the elevated glucose concentration in the drinking water. Hypertriglyceridemia may result from excess glucose consumption [18], and it has been reported that the rate of TG and fatty acid clearance is suppressed by cytokines, notably TNF-α, IL-6, and IL-1β, increasing this condition, as our data show [42]. In this paper, we analyzed the role of some inflammatory pathways, including those mediated by COX-2 and TLR4. COX-2 catalyzes the conversion of AA to prostaglandins and thromboxane [43], and this acid is the result of the desaturation of linoleic acid. These lipid mediators play important roles in inflammation and vasomotricity [44]. We found that COX-2 was increased in vessels from rats that received sucrose during the CP. There is an interaction between the COX-2, iNOS, and eNOS systems. We found a decrease in iNOS and a decrease in eNOS expression in aortas from rats that received sucrose during the CP. COX-2 metabolizes AA to PGE_2_ and is associated with changes in cytokine expression, such as TLR4, which is a pattern recognition receptor that plays a central role in the innate immune response. Activation of endothelial TLR-4 contributes to vascular inflammation [44]. TLR-4 expression is activated by monosaturated fatty acids such as oleic acid, and it can also be triggered by OS through oxLDL and oxidized phospholipids, with the participation of nuclear factor kappa B [45]. OS is increased in the CP window [28]. Activation of TLR4 induces nuclear factor kappa B activation and COX-2 expression [24]. Moreover, disturbed blood flow in arterial branches and curvatures causes shear stress, modulates endothelial function, and predisposes the region to endothelial inflammation, possibly through the participation of TLR4 [44]. Alterations in TLR4 activity can exert control over the expression of pro-inflammatory cytokines like IL-1β, IL-6, TNF-α, and type 1 interferons [46]. We found that TLR4 expression and oleic acid levels were increased in CP, and this could indicate the participation of an important function of this protein in the inflammation process in the CP (Figure 3, Table 1).

It has been reported that lipids have different biological functions and that changes in their concentrations can be linked to the secretory program associated with senescence, which consists of the production of pro-inflammatory factors and extracellular matrix remodeling factors [47]. Particularly, hypertriglyceridemia is an independent risk factor for cardiovascular disease and is linked to vascular dysfunction and an exacerbated inflammatory response [35]. In this sense, oleic acid contributes to increasing SBP through the inhibition of the eNOS pathway and favors an increase in iNOS. Our results showed that this fatty acid was increased in the CP group, and this could be associated with the increase of systolic blood pressure. Additionally, the iNOS and COX-2 pathways may contribute to the remodeling of the vessels because these enzymes are overexpressed by anatomical changes in VSMCs, and this contributes to inflammation and an increase in SBP (Figure 2). Our results showed a tendency to increase and an elevation, respectively, in the CP group. This suggests that the CP is very important because the physiological and anatomical changes in the vessels begin to appear in these animals due to the early insult of carbohydrates and remain marked and susceptible to consequences that appear later with age with a possible more aggressive response.

In this paper, we explored if inflammatory mediators such as TNF-α, IL-1β, and IL-6 were modified in the aortas from CP rats (Figure 4). In this sense, TNF-α is involved in the shift from a contractile to a secretory phenotype of VSMCs. This switch promotes proliferation and production of extracellular matrix proteins, which are associated with hypertrophy of the medial [16] and seems to be present in our group of CP rats, since α-actin (SMA), a marker of this shift, was decreased (Figure 5). The expression of TNF-α in the aortic tissue in CP rats can lead to the expression of SMA. TNF-α induces growth and/or migration of VSMCs and stimulates the production of chemokines, leading to a ‘proinflammatory’ phenotype of VSMCs. As previously mentioned, TNF-α was significantly increased in the CP group, and there was a clear tendency for IL-1β also to be increased. Both these inflammatory mediators promote the secretion and expression of other cytokines and cell adhesion molecules, including IL-6 [19], which was increased in the aortas from rats that received sucrose during the CP. Furthermore, it has been documented that the increase in shear stress accelerates the proliferation and turnover of endothelial cells, promotes the inflammatory response, and adjusts the vascular SMC phenotype towards the secretory phenotype [48]. In this paper, we found that smooth muscle β-actin was decreased in aortas from the group of CP rats. A decrease in smooth muscle β-actin is a marker of the media layer remodeling to a secretory phenotype, which could underlie a decrease in contractility leading to hypertension. Although α-actin (SMA) is the contractile protein and the one that, if decreased, acts as a marker of the secretory phenotype, β-actin is also present in VSMCs, forming part of the “cytoplasmic”, i.e., cytoskeletal “non-muscle” actin [49]. We also determined the expression of β-actin and found that ti was also decreased in CP rats.

There are reports that demonstrate that elevated SBP and structural modifications of the vessels are associated with the presence of OS, low bioavailability of NO, and levels of MMPs [50]. In this sense, a study previously published by our group [10] demonstrated that the aortas of CP animals present OS and low expression of eNOS. Although this paper (Figure 2) only a tendency of eNOS to be diminished in the immunohistochemistry, we previously found that this enzyme was also diminished in the aortas from CP rats in western blot analysis [29] and can be associated with the morphological changes that we observed.

Inflammatory mediators can modify the expression of MMPs that results in changes in the phenotype of VSMCs [16]. The shift in the phenotype of VSMCs from contractile to secretory is characterized by modifications in MMP expression or activity. MMPs are endopeptidases that degrade proteins in the extracellular matrix, such as collagen and elastin. MMPs also modify endothelial cells, determine migration and proliferation of VSMCs, and alter Ca^2+^ signaling and contraction. Changes in MMP-2 and -9 underlie arterial remodeling, determining pathological disorders that include hypertension [28,51,52]. In this paper, we determined the expression of metalloproteinases (MMP-2 and -9) and found that there was a tendency for an increase in their expression in the aortas of CP rats (Figure 6). This increase, while not reaching statistical significance, could be due to the activity of the enzymes rather than to the expression, which suggests that the alterations we found in the aortas cannot be attributed solely to the presence of these enzymes, but it would be interesting to evaluate their activity. MMP-2 and -9 are gelatinases produced by several vascular cell types, including endothelial cells, pericytes, podocytes, fibroblasts, and myofibroblasts. They participate in type IV collagen degradation, vasculature remodeling, angiogenesis, inflammation, and atherosclerotic plaque rupture. MMP-2 is expressed constitutively on the cell surface, and it is involved in hypertension induced by maladaptive vascular remodeling by degrading extra- and intra-cellular proteins. In contrast, the secretion of MMP-9 is induced by external stimuli and is stored in secretory granules [53].

**Figure 8:**
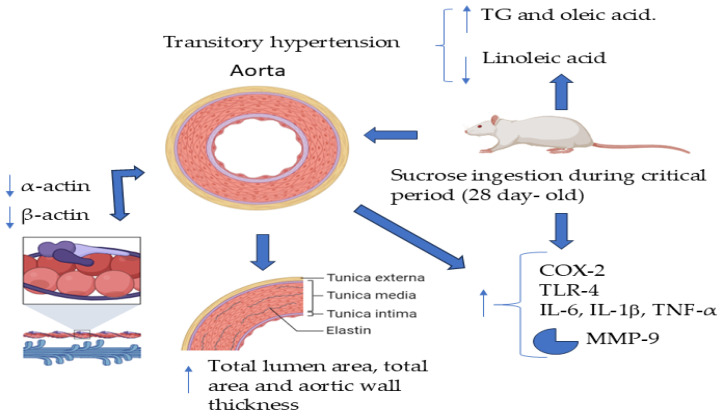
Excess consumption of sucrose in drinking water during the CP of vessel development in rats results in transitory hypertension accompanied by a change in the phenotype of VSMCs to the secretory type. There is a decrease in smooth muscle of β- and α-actin, which could act as an epigenetic cue to determine the development of hypertension when the animals reach adulthood. This change in the phenotype might be induced by increased inflammation characterized by increased levels of TNF-α, IL-6, and an increase in the expression of COX-2, TLR-4, and MMP-9, arrow-up = increase, arrow-down= decrease.

It is difficult to apply the results from basic studies in experimental animals on the possible role and mechanisms of changes induced in early stages to the programming of complex diseases including hypertension in humans. However, increased knowledge on these issues could help prevent the appearance of diseases if it is proven that the same mechanisms participate in human populations and could help prevent the appearance of the disease in adulthood. Figure 8 summarizes the findings described in this study.

## 5. Conclusions

In conclusion, when animals are exposed to sucrose in their drinking water during the CP of vessel development in rats, there is transitory hypertension accompanied by a change in the phenotype of VSMCs to the secretory type. There is a decrease in α- and β-actin. This change in phenotype might be induced by increased inflammation characterized by elevated TNF-α and IL-6 due to excess sucrose, which signals an increase in the expression of COX-2 and the TLR4 pathway, which are involved in the change in expression of VSMCs.

## Figures and Tables

**Table 1:** Body characteristics and biochemical parameters in the C group receiving the normal diet and rats receiving the sucrose diet during the CP.

	C	CP
Body Weight (g)	128.3 ± 1.5	105.2 ± 2.2 *
VAT (mg)	453.8 ± 0.05	460.7 ± 0.07
Glucose (mg/dL)	60.5 ± 3.9	63.0 ± 3.3
Insulin (µU/mL)	0.93 ± 0. 04	0.85 ± 0. 07
HOMA-IR	0.149 ± 0.047	0.102 ± 0.06
Triglycerides (mg/dL)	74.99 ± 3.58	120.93 ± 10.38 *
Oleic Acid (%)	17.88 ± 2.69	22.93 ± 0.77 *
Linoleic Acid (%)	15.18 ± 0.69	12.19 ± 0.40 *
AA (%)	8.55 ± 0.70	7.40 ± 0.61
SBP (mmHg)	93.63 ± 0.63	107 ± 0.10 *

Abbreviations: AA = Arachidonic Acid, VAT = Visceral Adipose Tissue, SBP = Systolic Blood Pressure * *p* < 0.05 vs. C, *n* = 8 animals per group. Values represent the mean ± SE.

## Data Availability

The datasets generated and analyzed during the current study are available from the I.P.-T. and V.C.-T.
